# Exploring the Applicability of Case Study Research in Investigating Service Integration

**DOI:** 10.5334/ijic.9818

**Published:** 2026-07-14

**Authors:** Dominika Lisiecka, Edelweiss Aldasoro, Niamh Lennox-Chhugani, Ingo Meyer, Adriana Poppe, Áine Carroll

**Affiliations:** 1 International Foundation for Integrated Care, United Kingdom; 2 Munster Technological University –Kerry Campus, Faculty of Health and Social Sciences, Ireland; 3 PMV research group, Medical Faculty and University Hospital Cologne, University of Cologne, Germany; 4 University College Dublin, Dublin, Ireland; 5 National Rehabilitation Hospital, Dublin, Ireland

**Keywords:** case study research, integrated care, co-creation, palliative care, health service evaluation, mixed-methods

## Abstract

**Introduction:**

The primary focus of this paper is the application of case study design to investigate the integration of complex health services.

**Description:**

This paper presents a co-created case study research project that examined the integration of a regional palliative care service. It outlines the study’s design including the mixed-methods approach to data collection and the processes used for analysis and triangulation. The paper also details the tools and strategies implemented across the macro, meso, and micro levels of the service, offering practical insights into how case study research can be applied to explore the dynamics of integrated care.

**Discussion:**

Case study methodology enables comprehensive, context-rich data collection and supports theory development through real-world observation. Its adaptability makes it particularly suited for evaluating complex systems. Co-creation added significant value by fostering interest-holders’ engagement and enhancing the relevance and applicability of the findings. The iterative and flexible design also allowed the study to respond effectively to challenges such as COVID-19 restrictions and a national cyber-attack.

**Conclusion:**

Case study research is a robust and flexible approach that provides deep insights into the integration of complex healthcare systems. Its co-creation potential and adaptability make it particularly useful in real-world health service evaluations.

## Purpose

This paper aims to provide a detailed account of a case study research project investigating the development, evolution, success, and transferability/replicability of an integrated regional palliative care service. The purpose is to provide methodological guidance for applying case study research for integrated care service evaluation.

## Focus

The focus of this paper is on how the case study research methodology can be applied to evaluate integrated care services and present the methods and types of data triangulation developed through co-creation process to ensure in-depth data collection and metaperceptual data analysis.

## Introduction

The growing recognition of healthcare systems as complex socio-ecological-technical systems has highlighted the limitations of traditional research approaches in understanding how change unfolds in practice [1]. These systems are shaped by dynamic interactions among people, technologies, institutions, and environments, evolving through non-linear, adaptive processes that resist prediction and control. Traditional methods, while valuable, fall short in understanding and responding to these complexities. As health systems confront mounting pressures, from demographic shifts and workforce challenges to digital transformation and climate disruption, there is an urgent need for research approaches that embrace complexity, attend to relationships, and make sense of emergence within interdependent, context-sensitive settings.

Case study research is a method of empirical social research that enables a detailed and nuanced investigation of a current or contemporary phenomenon [2,3,4]. Yin defined a case study research as an empirical method investigating a phenomenon of interest (the case) in detail and “within its real-life context, especially when the boundaries between phenomenon and context may not be clearly evident” p. 15 [3]. Case study research provides insights into causal mechanisms and intervention effects and is particularly useful for researchers and policymakers seeking to understand how programs work in specific contexts [5]. However, challenges exist, including misconceptions that case study research is only exploratory, a lack of consensus on what constitutes case study research, and difficulties in extracting key evaluation messages due to its detailed, context-rich nature [5]. Clearer methodological guidance is needed for reporting case study research effectively to maximise its impact in health services and public health research [5].

Palliative care is a person-centred, context-dependent, and multidisciplinary [6] complex adaptive system consisting of a variety of elements or “agents” interacting in a non-linear way [7]. Complexity theory helps us understand how different people and parts of the system work together, change over time, and respond to new situations. It focuses on connections, patterns, and how small changes can lead to big effects, which is useful for understanding how palliative care really works in practice [7]. These characteristics align well with the advantages of a case study research approach, which offers breadth, a collaborative nature, recognition of complex contexts, utilisation of multiple research methods, a realistic focus on process and outcome, and a linear but iterative methodology [4]. Although the value of case study research in palliative care has already been reported [8,9], there still is a relative invisibility of this approach in palliative care research, especially as a method for investigating a service integration or integrated palliative care services. This suggests that researchers interested in evaluating integrated palliative care services risk overlooking this potentially valuable and impactful research approach. To advance health evaluation research beyond current limitations in methods for understanding interventions as disruptions within complex systems, greater attention must be given to how researchers can effectively conduct and report case study research to uncover the contextual factors influencing interventions and their outcomes. Case study research is recognised to benefit from having multiple sources of evidence to allow the researchers to address a broader range of historical and behavioural issues, and a mix of qualitative and quantitative data allows for triangulation [4]. Carter et al. (2014) define triangulation as a key strategy in qualitative research that involves employing multiple methods or data sources to enhance the depth and validity of a study [10]. By integrating diverse perspectives, researchers can achieve a more comprehensive understanding of the research problem and ensure credibility through the convergence of information from various sources. In qualitative research, triangulation enhances the depth of understanding of a given phenomenon, and it is also employed to strengthen the credibility, validity, and overall trustworthiness of research findings by integrating multiple sources or methods [11].

When incorporating mixed methods into an evaluation, careful consideration of the sequencing of different approaches is essential. Harrison et al. (2020) provide a structured typology of mixed methods and corresponding strategies, guiding evaluators to determine whether these methods should be implemented sequentially or concurrently based on the specific evaluation questions [12]. This approach ensures that the research design aligns effectively with the research objectives [13]. To this end, supported by the UK’s Medical Research Council work on complex interventions [14], we have embraced the challenge of implementing the case study research to evaluate a complex intervention, such as the integration of a palliative care service. Our aim was to understand the development and evolution of the service, identify the factors that explain its success and identify the potential for replicability and transferability to other integrated care settings.

## Methods

Our case was identified as the whole regional palliative care service including inpatient, outpatient, and community services. We applied a case study research methodology with mixed-methods approach, and data were collected at three levels: 1) macro: policy and strategy, 2) meso: local context including service planning, funding and governance, and 3) micro: the experience of the service user, family and representatives of the multidisciplinary team (MDT). Primary data sources were qualitative interviews, observation of the MDT meetings, journal entries, fieldnotes, the SCIROCCO self-assessment survey [15] and a participatory workshop. Secondary data sources were routine data on service use, review of the international literature, documents and physical artefacts provided by the service. Government guidelines in relation to COVID-19 restrictions were fully adhered to throughout the data collection process. This included social distancing measures, hand hygiene and wearing appropriate personal protective equipment. A detailed process was developed to avoid coercion and minimise the burden of research for participants. Ethical approval was originally granted on 17/02/2021 by the University College Dublin Human Research Ethics Committee (reference: LS-21-13-McGovern) and subsequent modifications to the research protocol were reassessed and approved on 15/10/2021.

**Figure 1:**
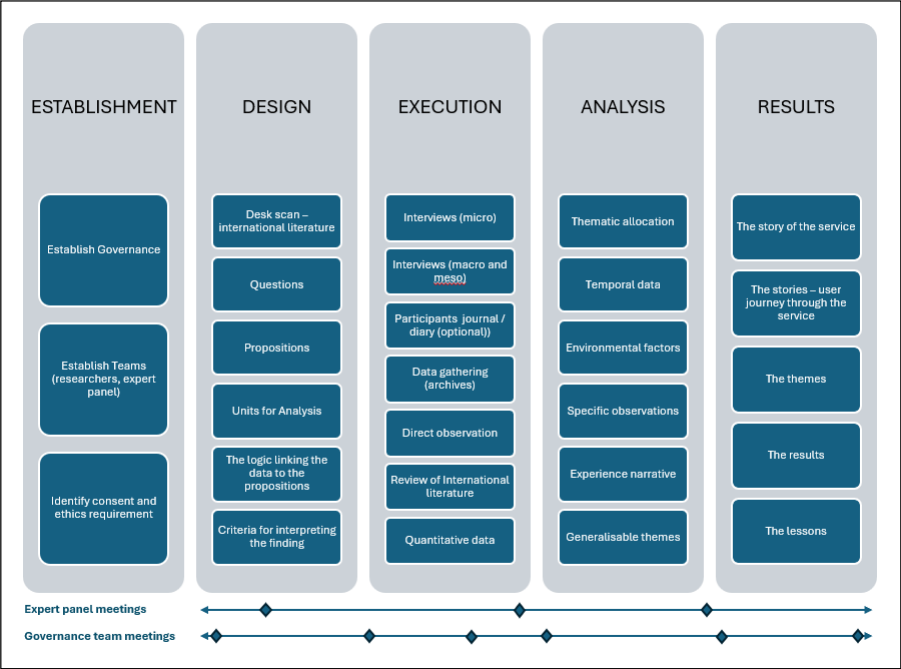
Description of the project plan.

**Figure 2:**
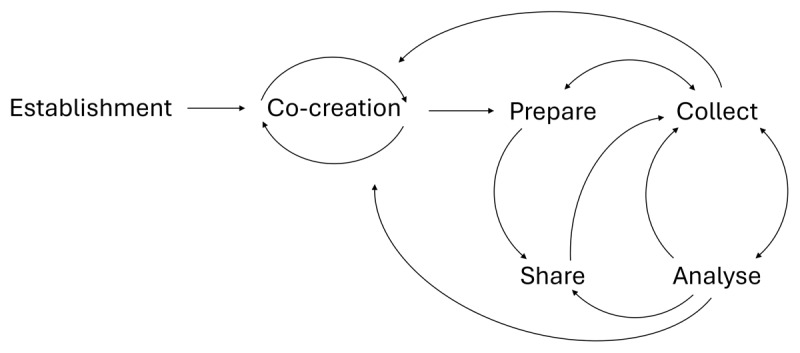
An iterative process of the case study research approach.

Methods are further summarised in Table 1. The recommendations of the Methods of Researching End-of-Life Care (MORECare) project [16] were considered in the development of the research evaluation project. An overview of the project plan is presented in Figure 1, followed by a graphical representation of the iterative process of the case study research approach (Figure 2). Figure 2 is adapted from Yin (2009) [17] to illustrate how our study extended the conventional case study approach by embedding principles of co-creation and continuous iteration with the governance group. While Yin’s original model presents a more linear progression between planning, design, preparation, data collection, analysis, and sharing, our adaptation emphasises the cyclical and participatory nature of the research. Specifically, it integrates a co-creation phase and multiple feedback loops between data collection, analysis, and dissemination activities, reflecting the collaborative and reflexive structure present at all stages of this mixed-methods evaluation.

**Table 1:** Methods and levels of data collection and sample sizes.


METHOD	MACRO	MESO	MICRO

Semi-structured interview conducted online or in person	National policy representative N = 1	Hospice and palliative care service representatives, N = 2	Total: N = 15 N = 3 service users currently accessing the service for ≥ 4 weeks N = 3 family members of people currently accessing the service for ≥ 4 weeks N = 3 family members of people who accessed the service in the past (now deceased) N = 6 MDT members

Observations– in person			Observations of MDT meetings N = 3

Reflective journal			Service users, their families and MDT

Fieldnotes			Research team

Service documents and artefacts	Newspaper articles, photos, letters, e-mails, annual reports, service plans, agendas, meeting minutes and other administrative documents provided by the service and the family of the service founder	Newspaper articles, photos, letters, e-mails, annual reports, service plans, agendas, meeting minutes and other administrative documents provided by the service and the family of the service founder	

Survey and workshop – online	National representative N = 1	Service providers and management team, N = 11	

Service metrics (routine data)			Key Performance Indicators plus individual level service use data: last year of life for all service users who died in 2019 and 2020 (N = 1727) incl. demographic, referral source and reason, hospital admissions, time using syringe driver/infusion pump, place of death, any other services used, and other service experience measures collected.

Literature review	International perspective and context		


### Co-creation with the interest-holders

The purpose and objectives of the evaluation, the case study research approach and the selected methods were co-created with the interest-holders and contrasted and validated with international experts on integrated palliative care service delivery. A stakeholder mapping exercise was conducted to identify individuals who should be involved across macro, meso, and micro levels. We defined “interest-holders” as groups with legitimate interests in the health issue under consideration [18]. We defined co-creation as collaborative knowledge generation by academics working alongside other stakeholders: representatives from the national health service, the volunteer-led organisation supporting local palliative care services, and the regional palliative care service. This coalition had existed since the inception of the regional palliative care service. The volunteer-led organisation included individuals who used the service and their families, community members, and local health and care professionals. Their perspectives on service development are gathered through regular meetings, and these contributions help inform and guide future service development. They contributed a community-informed perspective by bringing together members of the community with an interest in the development of palliative care services for the local population. Some members of the co-creation group were also research participants.

### Governance group

From the beginning, an interest-holders mapping exercise identified the partners who should be involved in the governance group. The project governance group oversaw the project throughout its duration and, upon completion, participated in discussions on how to best apply the findings and recommendations locally, identify aspects that could be adapted nationally, and determine the most effective ways to disseminate the information. The constituency of the governance group was balanced between executive, operational delivery, policy, research, clinical input, and community representation through the volunteer-led organisation. The members of the governance group participated in a workshop at the end of the project in order to validate and reflect on the findings of the evaluation.

### Involvement of people with lived experience

Current service users or their families were not directly involved in setting research questions, designing the methodology, conducting the research, or interpreting findings as research partners. Their involvement in this study was primarily as research participants.

### Expert panel

An expert panel was constituted with a mix of international and local experts in the area of integrated palliative care. Their role was to review the methods and the results at checkpoints and provide a reflection on how the results relate to the broader international context of palliative care. The outcomes of expert panel meetings were included in the data analysis.

Data were collected in person and online, as the research team members were based across Ireland, the UK, Germany, and Spain. The study was conducted during the COVID-19 pandemic, and during the data collection process, the national health service suffered a cyberattack [19]. This caused some delay and disruption to the project, predominantly as the participants had difficulties connecting online and accessing their emails. However, no changes to the study design were required.

#### Interviews

At the macro and meso levels, the interviews were tailored to the expertise and background of the interviewee (Appendix 1). Some of the focus areas included:

– Historical and contextual factors that led to the development of the service
– Care integration
– Milestones and challenges of the journey
– Governance structures and leadership
– Evaluation, progress and impact
– Digital solutions
– Finances
– Sustainability

At the micro level, the interview questions (Appendix 2) were adapted from the 59 key thematic areas identified for integrated primary care [20]. All participants were offered the opportunity to review their transcripts and check for accuracy before the analysis took place.

#### Observations

Observations of three MDT meetings were conducted to understand the context of the service and provide insight into the nature of MDT meetings. A data collection template was developed based on the same thematic areas as for interviews [20]. The template was piloted during the first observation and subsequently modified (Appendix 3). No individual identifiable data were collected during the observations.

Data from in-depth interviews and observations were complemented by the researcher’s fieldnotes and reflective journal entries recorded throughout data collection. These notes supported data analysis alongside interview transcripts and observation recordings. Participants were also invited to record their own reflections, although this was optional.

#### Service documents and artefacts

Data were collected from the following documents and physical artefacts:

Multiple sources were used, including peer-reviewed journal articles published by the service, national reports referencing the service, and other documents from commercial publishers (e.g., newspapers). Additional data were drawn from grey literature (e.g., internal reports, working papers), grey data (user-generated online content such as social media), and grey information, including both published and unpublished materials (e.g., annual reports, strategy documents, meeting notes, emails, and personal records).

#### The survey

The SCIROCCO Exchange Self-Assessment Tool is a questionnaire developed to assess the readiness for integrated care [15]. The tool was used to meet the research objectives: 1) understand the strengths and weaknesses related to the service integration; 2) facilitate the interest-holders dialogue about the implementation and delivery of integrated care; and 3) inform the service scalability and transferability. The tool had three steps: 1) the interest-holder mapping, 2) the SCIROCCO tool, and 3) the workshop for interest-holder dialogue. The interest-holders mapping exercise identified the relevant interest-holders from the macro (national representatives), meso (organisational management and service leads) and micro (care services and practitioners in all settings of the service) levels. Based on this exercise, the sample size (n = 12) was estimated.

The SCIROCCO tool assesses the maturity of the service in relation to 12 dimensions (Table 2). The population approach was eliminated from the exercise in agreement with the participants because it was not within the scope of the service evaluation. The Funding dimension was adapted to better reflect the context of the service.

**Table 2:** Dimensions of the Maturity Model of the SCIROCCO Exchange Tool.


MATURITY MODEL

1. Readiness to Change

2. Structure & Governance

3. Digital Infrastructure

4. Funding

5. Process Coordination

6. Removal of Inhibitors

7. Population Approach

8. Citizen Empowerment

9. Evaluation Methods

10. Breadth of Ambition

11. Innovation Management

12. Capacity Building


Following completion of the individual exercise, participants were invited to an online sensemaking workshop, facilitated by a member of the research team, to discuss domain-level results and reach consensus. The workshop aimed to explore stakeholder experiences across domains, build shared understanding, strengthen relationships, identify service strengths and challenges, assess domain maturity to inform scale-up or transfer, and establish baseline results for future comparison.

#### Service metrics (routine data)

Two types of routinely collected service data were used: Key Performance Indicators (KPI) data (2018–2020) and anonymised individual-level service-use data. The anonymised dataset was compiled from multiple sources, including in-patient, day unit, community records, and hospital information systems, with data collection overseen by a dedicated administrator to ensure accuracy. This dataset included demographics, diagnoses, and service use in the last year of life (e.g., referrals, admissions, services accessed, and place/date of death) and was subsequently analysed and reported by the data analyst.

#### Literature review

A preliminary scoping literature review was conducted to inform the evaluation and situate findings within a global context. Both peer-reviewed and grey literature were included, with a systematic search identifying 2,204 studies, of which 64 underwent full-text review. Eighteen studies provided relevant data on integrated palliative care models and service pathways.

## Data Analysis

The data analysis incorporated three stances – philosophical, strategic, and integrative – considered concurrently rather than in a linear sequence. Researchers engaged in reflexive analysis and inter-disciplinary learning, acknowledging how their ontological and epistemological beliefs influenced knowledge production.

A key analytical technique was data triangulation, which involved developing converging lines of inquiry to enhance the reliability of findings across multiple data sources. The validity of the findings, particularly in establishing cause-and-effect relationships, was ensured through agreement among different data types, systematic elimination of alternative explanations, and examination of outlier results. Consistency of evidence across diverse data sources served as a means of verification.

Qualitative analysis was guided by framework analysis [21] and followed a structured, iterative process. Transcripts were read repeatedly to achieve familiarisation, followed by inductive open coding to capture emergent insights. Themes were then refined in line with the research aims, and a subsequent deductive phase categorised the data according to predefined themes based on the nine pillars of integrated care.

This combined approach was appropriate given the complexity of the data and the project’s aims and objectives. NVivo 12 was used for data management and coding. Regular meetings of the research team took place to present and discuss the themes emerging from the data.

Deductive content analysis was used to identify and analyse the information from documents and physical artefacts to understand the journey of the service and its value in the community [22]. The data were analysed in line with the research aims: development, evolution, success, replicability and transferability.

The results of the literature review were discussed and validated with an international palliative care expert panel.

The service metrics data were separated by service type (outpatients, acute, community). To track the development of individual indicators for each service, total values were calculated annually. Where applicable, percentages were used to compare different characteristics of the indicators (e.g., cancer vs. non-cancer). Each service type was assigned a unique ID, which allowed for linking entries from different claims to individual patients. This enabled tracking a patient’s trajectory across and within different services. The data was then imported into STATA 15 for analysis.

To assess data quality, plausibility checks were conducted. For example, it was verified whether different genders were assigned to the same patient. The results of these checks revealed discrepancies in gender, place of death, and age at death for some patients. Since it was not possible to determine the correct information from the available data, these inconsistencies were recoded as “unclear” and the data were then included in the analysis.

The data were analysed descriptively. Frequency distributions of the basic variables (e.g., gender, diagnosis, place of death) were calculated, and cross-tabulations were performed. Additionally, the number of days between referral and the first visit, as well as the outcome, were calculated. Finally, the number of services received by patients in the 365 days prior to the outcome (death) was determined, organised by individual Consultant Palliative Day Outpatient Clinics.

We used pattern matching, explanation building, and thematic analysis, employing techniques such as graphic data displays, tabulations of event frequencies, and chronological or time-series orderings. This approach allowed us to achieve four types of triangulation [10]:

1. Data triangulation (from different data sources, which helps address issues of construct validity)
2. Investigator triangulation (across different members of the research team)
3. Theory triangulation (by applying various perspectives to the same data set)
4. Methodological triangulation (by using multiple data collection tools)

During the data analysis process, regular meetings of the research and governance team took place to discuss the emerging findings.

### Positionality and Reflexivity

The research team approached this study from the belief that multiple perspectives are essential to understanding complex systems such as palliative care. Informed by a constructivist stance, we recognised that knowledge about integrated care is co-created. Our positions as clinicians, policymakers, and researchers inevitably shaped data interpretation and conclusions. Team members occupied both insider and outsider roles: some brought professional experience in service delivery and health system leadership, while others contributed academic expertise in health systems research and participatory methodologies. This diversity required continual reflexivity to surface and examine assumptions. Reflexivity was embedded across the research cycle through team discussions and engagement with the governance group. For example, during analysis of interest-holders workshop data, team members acknowledged that their own backgrounds influenced how they interpreted tensions between statutory and voluntary perspectives. Reflecting on these interpretations with the governance group enabled us to refine coding decisions and arrive at conclusions that more accurately reflected the multiple realities of participants.

## Findings

Case study research was beneficial in providing detailed insights into the service integration. Despite the challenges of the pandemic, the national cyberattack, and the extended project timeline, our case study approach allowed for continuous stakeholder engagement, iterative reflection, and adaptation of our research process – demonstrating the practical value of case study methodology in complex and evolving systems. The specific results related to the integration of the local palliative care service are published elsewhere.

Having the service representatives involved in co-creation of the study provided an opportunity to discuss key decisions and greatly contributed to collecting comprehensive data. The mixed-methods design enabled in-depth investigation of the service as a complex system. Multiple perspectives and data sources allowed data triangulation and contributed to the validity of findings. The expertise of the members of the Governance Group and Expert Panel enabled an additional layer of validation for the final results.

By focusing on specific instances of integrated care implementation, case study research can reveal the nuances of service delivery, coordination among care providers, and the experiences of patients and service users. For example, through sustained engagement with stakeholders we were able to highlight the impact of local leadership and historical relationships with the local community on integration success, which emerged through narrative and timeline mapping exercises.

These types of findings demonstrate how case study research can capture the complexity and contextual nuances of integrated care that are often difficult to expose using other methodologies.

## Discussion

In line with the purpose of this paper, we do not present the results related to the regional palliative care service itself. Instead, our key finding is that a case study is a suitable methodology to evaluate a complex palliative care service in its entirety. This methodology allowed for iterative, flexible, and adaptable method of data collection, which accommodated the challenges experienced during this service evaluation (such as changing national restrictions related to COVID-19 or access to virtual space due to a cyber-attack). Case study research evaluations excel in providing contextualised insights into the phenomena under study. It allows researchers to collect comprehensive and context-rich data, which can provide a deeper understanding of complex issues that may not be easily captured through other methodologies [2]. For example, in health research, a case study can offer a detailed account of patient care experiences, uncovering subtle nuances and deeper insights that quantitative methods might overlook, and thereby offering a comprehensive understanding of the case [23]. Case study research contributes to theory development by generating new concepts or hypotheses based on real-world observations [24]. This is particularly valuable in fields like healthcare, where theoretical models may need to be adjusted to account for the complexity of real-world settings.

Although case study research methodology is commonly employed in health research, it has played a limited role in evaluative studies due to the prevailing belief that case studies are not useful for drawing causal conclusions [25]. Many evaluative studies, especially in the context of complex systems, require flexibility of approach. Case study research is adaptable to various research settings, enabling the study of single or multiple cases. It also allows researchers to use a mix of data collection methods (e.g., interviews, observations, documents) and to refine their questions as new insights emerge [26].

Co-creation in case study research can offer significant advantages, particularly in terms of enhancing the relevance, validity, and applicability of the study [27,28]. In our study, co-creation strengthened access to contextual knowledge, supported dialogue across interest-holder groups, and improved the interpretive depth of the evaluation. These benefits are consistent with wider arguments that collaborative knowledge generation can increase the practical usefulness of research and its uptake in policy and service development [28].

Several limitations should nevertheless be acknowledged. First, although the study incorporated co-creation, the level and form of participation varied across groups and stages of the research. In particular, the representation of lived experience through a voluntary organisation provided an important community-informed perspective, but it could not fully substitute for direct engagement with a diverse range of service users and family members in the design and interpretation of the study. Second, as a single case study, the findings are inherently context dependent. While they offer analytical insights with potential for transferability, they should not be interpreted as universally generalisable without careful attention to contextual differences. Third, the breadth and volume of data required substantial interpretive work, which may introduce researcher bias despite the use of triangulation, reflexivity, and governance-based validation. Finally, the evolving nature of the service and the external disruptions experienced during the study, including COVID-19 and a national cyberattack, may have influenced both data collection and interpretation.

Our experience using a case study methodology confirmed many of the benefits and challenges discussed in the wider literature [5]. On one hand, we found that case study research is not as widely used or standardised in comparison to other research and evaluation approaches, and as such, reaching consensus on the definition, methodology and reporting required longer investment from the research team. On the other hand, the case study approach provided the frame to explore and understand the dynamic and evolving influence of the context in “real-life” and therefore, help us determine what can be transferable to other settings or contexts. It was also considered a valuable approach to understand organisational and policy-level contingencies that can affect the adoption and implementation of an initiative. Despite the lengthy duration of the project, engagement from both the research team and the service was maintained throughout, likely due to the co-creation approach, contextual factors, and existing collaborative structures within the palliative care service. The service operated with relatively small caseloads but high-intensity, relationship-based support, which may have reduced the risk of disengagement. Furthermore, the case study was embedded within well-established collaboration mechanisms. As the study aligned closely with routine service activities and values, participation was experienced as a natural extension of ongoing practice rather than an additional burden.

Based on our research, we propose that the case study research methodology is a suitable approach for supporting integrated care research, in particular with a view to evaluating complex, real-world service scenarios.

Findings from case study research can be used to refine or develop new policies that promote better integration across care levels, improving patient outcomes and optimising resource use [29]. Our experience suggests that case study research is a valuable methodology applicable to investigating integration of complex healthcare services. As such, case study research is an essential tool for advancing integrated care research, offering practical insights that can influence policy development and improve healthcare delivery.

## Conclusions

Case study research offers a methodologically robust approach for investigating the integration of complex health and care services within real-world contexts. Its strength lies in its ability to capture interactions among system components, actors, and environments over time, generating insights often inaccessible through more reductionist methodologies.

This project demonstrates that case study research can support a deeper understanding of how integrated care develops, operates, and adapts under conditions of uncertainty. It is particularly valuable for examining the interplay between organisational, professional, policy, and community factors, and for identifying barriers and facilitators that influence the implementation of integrated care in practice.

Our findings also reinforce the importance of transparency in how co-creation is conceptualised and reported. Co-creation can enhance contextual relevance, engagement, and interpretive depth, but its value depends on a clear description of who was involved, how, and at what stages of the research process.

Rather than positioning case study research as a merely descriptive approach, we argue for its role as an analytical and theory-informed methodology capable of informing policy, practice, and system transformation. When rigorously designed, co-created where appropriate, and transparently reported, it provides a valuable contribution to advancing integrated care research and evaluation.

## Additional File

The additional file for this article can be found as follows:

10.5334/ijic.9818.s1 Appendices. Appendix 1 to 3.
