# Supplementary material for: Exploring the Applicability of Case Study Research in Investigating Service Integration
Source: Int J Integr Care. 2026 Jul 14;26(3):5. doi: 10.5334/ijic.9818 (PMC13378430; doi:10.5334/ijic.9818)
Supplement: Appendices. — Appendix 1 to 3. [file ijic-26-3-9818-s1.pdf]

Appendix 1. Interview guide: Macro and Meso Level

Date: ___________ Interviewer: _________________ Interviewee: __________

1 Interviewer Script

1.1 Introduction

First of all, thank you so much for participating in this interview. My name is […], I am a researcher in IFIC and I am with the […].

These interviews are part of the case study research we are conducting to evaluate the [name service]. As you know, [name service] has been named as a good example of integrated palliative care service. It was created with the bottom-up approach, driven by the initiative of the local community and led by the local voluntary organisation. The shared vision and values in the different levels, from community leaders to the highly motivated care team, and supported by external authorities, have produced a unique and exemplar journey of a service development.

With these policy or system level and organisational level interviews we would like to understand the factors that explain the emergence and evolution of the [name service]. We would also like to know what went well, which were the challenges and barriers in this journey, how you overcome them and where you see the opportunity for applying this knowledge outside of [name geographical area]. Your perspective will also inform the project in identifying future steps and planning of the local service.

As explained by email, we will video record the interview, and we will get automatic captions. We will also take notes during the interview. Once we get the final transcription, we will delete the recording. Only the participants of this call will hear the recording, and any direct quotation will be anonymised.

You also know that your participation is voluntary, and you can refuse to answer any question or withdrawn at any time.

2 Consent

Do you have any further questions before we begin? Do you agree? Can we proceed?

3 Semi-structured dialogue – topics to cover

Theme	Questions	Probes
Icebreaking	Tell me the story of your involvement in the development of the [name service]	How did you know about the initiative in [geographical area]?

Shared goals	How did the local initiative fit with the national strategy?	Were national and local perspectives similar?
National level strategy	How has the interest in Palliative Care evolved at the national level?<br><br>How has the Palliative Care model matured in Ireland?<br><br>What was/is your care model and care pathway reference to design the Palliative Care model?	How did the [name service]fit in the national strategy?<br><br>How had [name service] progressed compared to the national level care model?<br><br>How much of the reference care model does the current [name service] fulfil and what is still under development or pending?
Relevance	Are there other experiences like [name service]in Ireland?	What makes [name service]unique in Ireland?
Milestones	What are the milestones in the development and journey of the [name service]?	** (see some examples of national documents at the end)
Governance	Where the governance structures and roles in the [name service] clear in the interaction with national level bodies?	Do you see any improvement areas in the governance of the [name service]?
Leadership	What kind of leadership did you observed in the development of [name service]?	Has the leadership model you observed had an impact in the development of the service?
Good practices	What are the ingredients of the [name service] model that make it different from the usual model of palliative care in Ireland?	Person-centredness<br><br>Personalised care

		Community involvement<br><br>Embedded in local culture<br><br>MDT<br><br>Care team support and professional development<br><br>Organisational coordination and partnership<br><br>Referral process and integration of the services<br><br>System coordination, including policy, financing and political commitment<br><br>Technical and digital competence: interoperable medical records, virtual care<br><br>Measurable outcomes and KPIs, including efficiency, cost, patient and family and care team satisfaction
Problem solving	What have been the main difficulties in the development of the [name service]?<br><br>What have been the main enablers to develop the service?	Can you think of an example of a problem that might have happened and how it was solved?
Provider	If the [name] Hospice had not existed, what	How do most Palliative Care

outcomes	would have meant for the provision of Palliative Care in [geographical area]?	services develop in other areas in Ireland considering the national strategy?
Evaluation	Based on the reference KPIs or measures that are relevant to assess and compare service delivery, how does [name service] perform?	Are there any indicators where [name service] should improve?<br><br>What kind of support would [name service] need to improve the indicators?
Sustainability and future	What are the challenges to make the service sustainable in the future?	Is the current model sustainable?
Future	Is there anything that you have learnt from [name service] that would inform the National Clinical Programme going forward?	Is this a model that you could see replicated in other parts of Ireland?<br><br>How would this replication happen?
Closing	Is there anything we have not asked you would like to share?	

4 Closing

Those are all the questions we have for you today. Thank you so much for your time. If you think of any other comments, feel free to contact me in my email. I will send you the transcription so you can have revise and add comments if you wish.

At the end of the transcription, we will add a space where you can add comments or notes about the interview that may not be reflected in the transcript.

5 Interviewer Reflection

(Include notes, non-verbal communication, issues for subsequent interviews, etc)

Appendix 2. Interview Guide: Micro level

FAMILY REPRESENTATIVE (CURRENT AND PAST)

Date: __________ Venue: _____________ Interviewer: _____Interviewee: ___________

Relation to SU: _______________SU' Diagnosis:___________________Group (1-7): _______

Living area: rural / urban / suburban Distance from service: ________

Way of access: ________________

Family situation:
____

Type of service: inpatient / community / day care / acute

Type of treatment: symptom management / palliative care / end-of-life care

Disciplines involved: ____

Two prof. most involved: _____________________________________

How long accessing service: ___________________Frequency: ______________________ Referred by:
____

Other services being attended:
____

Groups:

1 Non-malignant disease (e.g. MND) – experience of outpatients, day care, hospice, hospital and community care;
2 Non-malignant disease for EOLC. Short involvement in the hospital and referred to the community (home). May have required ICS night nursing etc;
3 Non-malignant disease for EOLC. Short involvement in the hospital and referred to the hospice for EOLC;
4 Group 2 or 3, represented by a family member
5 Malignant disease, experience of multiple aspects of the service – community, hospital and hospice;
6 A haematological diagnosis, experience of the service
7 Malignant disease transferred from the hospital or home to a community support bed (my need to be a family member for interview here also)

1. Introduction

First of all, thank you so much for participating in this interview. My name is [Name], and I am a researcher interested in learning about your experiences with the [Service Name] palliative care service. Our team aims to understand how the [Service Name] works from the perspectives of service users, family caregivers, and staff. [Service Name] has been identified as a good example of integrated palliative care, and we would like to learn more about how it operates from your point of view.

We are particularly interested in hearing what you feel works well within the service, as well as any challenges or barriers you or other service users may have experienced. Your insights will help us better understand the [Service Name] and apply this knowledge to support the development of similar services outside of [Geographical Area]. As explained earlier, I will be audio/video recording this interview. I may also take some notes during our conversation. Once the interview is transcribed, the recording will be deleted. Any names mentioned during the interview will be anonymised. If at any point you feel tired, please let me know — we can take a break or stop the interview. As you know, your participation is voluntary; you do not have to answer every question, and you can withdraw from the project at any time.

Consent

Do you have any further questions before we begin? Do you agree? Can we proceed?

2. Semi-structured dialogue – topics to cover

**Thematic areas**	**Primary questions**	**Secondary questions / cues**
**Previous knowledge of the service**	I would like you to go back in the past. What did you know about [name of service], before your family member became sick and was referred here?	Did you hear anything in the local media?
**Transition to the service**	How did your journey with the service begin?	Can you remember the first day at [name of service]?<br><br>I presume you were accessing other services before coming to the [name of service]. How was the transition from previous care teams to this service?<br><br>What do you think about the timing of your family member's referral to [name of service]? Was the care offered and available at the right time? Or was it too early or too late?
**Dimension: Clinical integration** (7 key features)		
1. **Centrality of client needs**	How much are (were) your family member's individual needs considered by the service?	Please think about all needs, not only medical.
2. **Service characteristics**	What services have (had) your family member received for his/her medical needs? What about social and psychological	How would you rate your satisfaction with the care and support received?<br><br>If you were to list three most positive features of

	needs?	the service, what would they be?
3. **Patient education**	What can you say about the education you and your family member have received from professionals?	Would you liked to have received more or less information?<br><br>How clear are you about the role of each professional in the service?
4. **Interaction between professional and client**	What can you tell me about the interactions you had with professionals?	How were your concerns acknowledged and understood?<br><br>In your opinion, how honest was the information provided to you?<br><br>How consistent was the information you heard from different professionals involved in your care?<br><br>How easy (or difficult) was it to contact the care team to ask for information?<br><br>How do you find the access to care team out of hours?<br><br>Have you experienced any delays with appointments?<br><br>Has there been any conflict between your preferences, your family's or the care team's? If so, how did the conflict solve? Did you feel there was enough support to solve the conflict?<br><br>Has anything changed in your interactions with professionals now during COVID?
5. **Information provision to clients**	How would you rate the clarity of information you have received from the professionals? How hard or easy has it been to understand the information? How do you feel your family member was satisfied with the information provided to him/her?	What type of communication have you received (face-to-face or virtually)?<br><br>Would you have preferred a different communication channel from the ones available?<br><br>What would be your preferred way of communication with the team?<br><br>What is your opinion about virtual consultations?
6. **Client participation**	What impact do you feel your family member have (had) on the care received?	How have your family member been involved in the decision-making about his/her care?<br><br>Do you think all the information was provided so you're your family member could make own decisions?<br><br>Have your family member's preferences always

		been respected and understood?
7. **Self-management**	What help have you received to enable your family member to self-manage some of his/her symptoms?	How important is the ability to self-manage some symptoms for you?
**Service improvement**	Do you have any suggestions about how to improve the service?<br><br>If you have a power to design the best palliative care service what would it be?	
**Closing**	Is there any other comment you would like to make?	Is there anything that I have not asked you about, but you want to share with me?

3. Closing

Those are all the questions we have for you today. Thank you so much for your time. If you think of any other comments, feel free to contact me.

I will send you the transcription so you can revise them and add comments if you wish. At the end of the transcription, we will add a space where you can add comments or notes about the interview that may not be reflected in the transcript. If easier, you can email your comments to me, or I can ring you and you can tell me about any revisions you wish to make on the phone.

4. Interviewer's reflection

(Include notes, non-verbal communication, issues for subsequent interviews, etc)

Appendix 3 Observation template

Meeting purpose	Attendees	Venue	Time	Chair

Agenda item	Time	People discussing	Comments

KEY FEATURES PER DIMENSION	MDT<br>Macro	Observed	Comment
Dimension 1: Clinical integration			
1. Centrality of client needs			
2. Case management			
3. Patient education			
4. Client satisfaction			
5. Continuity			
6. Interaction between professional and client			
7. Individual multidisciplinary care plan			
8. Information provision to clients			
9. Service characteristics			
10. Client participation			
11. Population needs			
12. Self-management			

Dimension 2: Professional integration			
1. Interprofessional education			
2. Shared vision between professionals			
3. Agreements on interdisciplinary collaboration			
4. Multidisciplinary guidelines and protocols			
5. Interprofessional governance			
6. Interpersonal characteristics			
7. Clinical leadership			
8. Environmental awareness			
9. Value creation for the professional			
10. Performance management			
11. Creating interdependence between professionals			
Dimension 3: Organisational integration			
1. Value creation for organisation			
2. Inter-organisational governance			
3. Informal managerial network			
4. Interest management			
5. Performance management			

6. Population needs as binding agent			
7. Organisational features			
8. Inter-organisational strategy			
9. Managerial leadership			
10. Learning organisations			
11. Location policy			
12. Competency management			
13. Creating interdependence between organisations			
Dimension 4: System integration			
1. Social value creation			
2. Available resources			
3. Population features			
4. Stakeholder management			
5. Good governance			
6. Environmental climate			
Dimension 5: Functional integration			
1. Human resource management			
2. Information management			
3. Resource management			
4. Support systems and			

services			
5. Service management			
6. Regular feedback of performance indicators			
Dimension 6: Normative integration			
1. Collective attitude			
2. Sense of urgency			
3. Reliable behaviour			
4. Conflict management			
5. Visionary leadership			
6. Shared vision			
7. Quality features of the informal collaboration			
8. Linking cultures			
9. Reputation			
10. Transcending domain perceptions			
11. Trust			

Reflective comments post-observation

Which attendees were most impacting upon decision-making actions?	
Were some speaking more than others?<br><br>Why?	

Were there attendees who were silent or contributed little to discussions?<br><br>What may explain their lack of contribution?<br><br>How was team dynamic overall?	
If this is a follow-up meeting, how have contributions from different attendees changed since the previous meeting?	
Was there any conflict observed?<br><br>How was it managed?	
How was the meeting chaired? What was effective/ineffective with regard to how the discussions were facilitated?	
Is there evidence of interprofessional collaboration?	
Was there clarification and evidence to support discussion?	
Did the meeting keep to time? If not, why?	
Any technology used?	
Did the meeting achieve its main objectives?	

Are there clear indicators of IC?	
Any other comments?	

Analytic questions: What do I see going on here? What did I learn from these notes? Why did I include them?
